# Nanotherapeutic approaches to overcome distinct drug resistance barriers in models of breast cancer

**DOI:** 10.1515/nanoph-2021-0142

**Published:** 2021-06-25

**Authors:** Tanmoy Saha, Jayanta Mondal, Sachin Khiste, Hrvoje Lusic, Zhang-Wei Hu, Ruparoshni Jayabalan, Kevin J. Hodgetts, HaeLin Jang, Shiladitya Sengupta, Somin Eunice Lee, Younggeun Park, Luke P. Lee, Aaron Goldman

**Affiliations:** Division of Engineering in Medicine, Brigham and Women’s Hospital, Boston, MA, USA; Department of Medicine, Harvard Medical School, Boston, MA, USA; Department of Electrical & Computer Engineering, University of Michigan, Ann Arbor, MI 48109, USA; Department of Biomedical Engineering, Biointerfaces Institute, Applied Physics, Macromolecular Science and Engineering, University of Michigan, Ann Arbor, MI 48109, USA; Department of Mechanical Engineering, University of Michigan, Ann Arbor, MI 48109, USA; Cancer Immunology, Dana Farber/Harvard Cancer Center, Boston, MA, USA

**Keywords:** cancer biology, chemotherapy, drug resistance, nanomedicine

## Abstract

Targeted delivery of drugs to tumor cells, which circumvent resistance mechanisms and induce cell killing, is a lingering challenge that requires innovative solutions. Here, we provide two bioengineered strategies in which nanotechnology is blended with cancer medicine to preferentially target distinct mechanisms of drug resistance. In the first ‘case study’, we demonstrate the use of lipid–drug conjugates that target molecular signaling pathways, which result from taxane-induced drug tolerance via cell surface lipid raft accumulations. Through a small molecule drug screen, we identify a kinase inhibitor that optimally destroys drug tolerant cancer cells and conjugate it to a rationally-chosen lipid scaffold, which enhances anticancer efficacy *in vitro* and *in vivo*. In the second ‘case study’, we address resistance mechanisms that can occur through exocytosis of nanomedicines. Using adenocarcinoma HeLa and MCF-7 cells, we describe the use of gold nanorod and nanoporous vehicles integrated with an optical antenna for on-demand, photoactivation at ∼650 nm enabling release of payloads into cells including cytotoxic anthracyclines. Together, these provide two approaches, which exploit engineering strategies capable of circumventing distinct resistance barriers and induce killing by multimodal, including nanophotonic mechanisms.

## 1 Introduction

Despite the risks associated with cytotoxic cancer chemotherapies, such as taxanes and anthracyclines, they remain a key part of treatment for more than half a century [1]. A developing paradigm to improve the delivery of drugs directly to tumors and reduce toxicity to normal tissue and cells is the blending of engineering with biology [2]. A variety of materials and technologies have been deployed to achieve this goal, which includes the use of lipids, polymers, inorganic carriers, hydrogels, and even plasmonic strategies that exploit thermal dynamics [3]. However, drug resistances have been shown to affect both the therapies themselves and the bioengineering strategies that are used to improve treatment response [4]. Therefore, rational development of engineered nanotherapeutics that harnesses discoveries in cancer biology and drug resistance may overcome many of these challenges.

Nanotherapeutics for cancer often harnesses protein and nucleic acid biomarkers to target payloads [5]. For example, decorating nanoparticles with aptamers, antibodies, proteins, and small peptides, such as arginylglycylaspartic acid (RGD), have shown improvement in reaching tumor cells and avoiding some of the toxicity associated with the cytotoxic payloads [3]. However, resistance mechanisms such as endosomal recycling and molecular biological signals that rely on cell survival pathways can limit the efficacy of these approaches [4]. For example, we recently made the discovery that dense lipid rafts are induced and accumulate on the surface of taxane-experienced cancer cells, which have phenotypically switched to a drug tolerant state [6]. This phenotype allows cells to circumvent cytotoxic chemotherapy through a mechanism that involves binding of caveolin-1, scaffolding of Src family kinase (SFK)/hemopoietic cell kinase (Hck), and translocation of nuclear proteins that inhibit apoptosis [6]. Separately, resistance to nanomedicines can manifest through extrinsic and physical barriers including endosomal recycling [7]. This unique mechanism of resistance has been challenged using membrane fusion, osmotic pressure, nanoparticle swelling and membrane destabilization to bind and disrupt the endosomal packages [8]. However, emerging approaches including plasmonics may provide novel opportunities to release drug payloads in a manner that potentially circumvents resistance via endosomal recycling [9]. Harnessing these discoveries to improve the uptake of anticancer drugs into subpopulations of refractory cells, circumvent molecular *and* physical barriers to treatment response to enhance cell killing is a critical milestone in drug development.

Here, we present two case studies in which the cell surface properties of cancer are exploited by rational engineering of lipid-based or plasmonic nanoparticles to overcome distinct mechanisms of resistance and drive anticancer effects. First, we describe the engineering of a nanotherapeutic based on lipid raft accumulation and molecular signaling pathways of resistance, which preferentially targets drug-induced resistant cancer cells to release a lipid moiety engineered with a kinase-inhibiting payload. In the second example, to preferentially force the release of drug payloads and thwart exocytosis leading to resistance, we engineer nanoparticles carrying activated payloads that release their contents via exposure to innocuous wavelengths of light, which overcome the physical intracellular compartments that can mitigate anticancer efficacy. Together, these results present engineering strategies that can target the biomolecular or physical barriers of resistance. Further interrogation of these approaches in rigorous *in vivo* and human proof of concept studies is warranted.

## 2 Materials and methods

### 2.1 Chemicals

Cetyltrimethylammonium bromide (CTAB), ethylene glycol (EG), ammonia solution, tetraethyl orthosilicate (TEOS), ammonium nitrate (NH_4_NO_3_), hydrazine (35 wt% in H_2_O), sodium azide, dimethyl sulfoxide (DMSO), phosphate buffered saline (PBS), Hoechst 33342, paraformaldehyde, and adriamycin were purchased from Sigma-Aldrich, MO, USA. 3-Aminopropyltriethoxysilane (APS) was purchased from Gelest, PA, USA. Nanopure deionized (DI) water (18.1 MΩ cm) was produced in house. 3-(4,5-Dimethylthiazol-2-yl)-2,5-diphenyltetrazolium bromide (MTT) was purchased from Thermo Fisher Scientific, MA, USA. NBD-Ceramide, NBD PC (810132). NBD PA (810176), NBD Cholesterol (810250), NBD PE (810144), NBD-PG, and NBD-PGPE have been purchased from Avanti Polar Lipids, USA.

### 2.2 Cell culture and generation of drug tolerant cancer cells *in vitro*

MDA-MB-231 (ATCC) were cultured in Dulbecco’s Modified Eagle Medium (DMEM) containing 10% fetal bovine serum, MDA-MB-468 (ATCC), SUM-159 (ATCC), MCF-7 (ATCC), HeLa (ATCC), and 4T-1 mammary carcinoma cells (ATCC) were cultured in DMEM or Rosewell Park Memorial Institute (RPMI) containing 10% fetal bovine serum (Invitrogen, Carlsbad CA, USA) at 37 °C and 5% CO_2_. Generation of drug tolerance was performed as follows: cancer cells were plated at a density of 0.5 × 10^5^ cells mL^−1^ and allowed to adhere for 24 h onto cell culture plates. When the cells reached ∼75% confluency, they were treated with the cytotoxic drug, Docetaxel, at indicated concentrations for 48 h and utilized for subsequent assays. Following washes with PBS, adherent cells were trypsinized and re-plated at a density of 2 × 10^5^ cells mL^−1^ and cultured in serum-containing medium. After 24 h incubation, the floating cells were removed and the remaining cells were washed with 1X PBS and considered to be the drug tolerant cancer cells (DTCCs). A population of drug naïve parental cancer cells (DNCCs) was always cultured alongside the DTCCs and fresh medium was added at the same interval that the DTCCs received fresh media.

### 2.3 Lipid raft imaging

DNCCs or DTCCs were generated as described above and plated in eight chamber glass slides (BD Biosciences, San Jose, CA) at a concentration of 10^5^ cells/mL. Before lipid raft analysis, cells were first exposed to fluorescent lipids (NBD-PC or NBD-cholesterol; fluorescent in the green spectrum) at the indicated concentrations. Subsequently, lipid rafts were labeled by Vybrant™ Alexa Fluor™ 594 Lipid Raft Labeling Kit according to manufactures protocol (ThermoFisher Scientific). Briefly, cells were washed with PBS and CT-B (Cholera toxin subunit B) has been added with basal media (2 μg/mL) and incubated for 1 h at 4 °C. Cells were washed 3 times with cold PBS and anti CT-B (5 μL/mL in basal media) has been added to that. After incubation for 30 min at 4 °C, the cells were fixed with 4% paraformaldehyde for 20 min. Cells were stained with 4’,6-diamidino-2-phenylindole (DAPI) and imaged by fluorescence or confocal microscopy. Fluorescent images were obtained using three channels on an NIKON Eclipse TI-U microscope with a 20× ELDW, 10 or 40× Plan-Apo objective lens (Nikon, Melville, NY). NIS Elements Viewer version 3.22 (Nikon, Melville, NY) software was used to capture the images to file.

### 2.4 Lipid uptake by flow cytometry

Cells were cultured as indicated, exposed to fluorescent lipids for the indicated amount of time, and washed twice with PBS. Cells were then processed by flow cytometry to analyze fluorescent intensity of cells (Accuri C6, Bectin Dickinson Biosciences). Data analysis was performed using FlowJo software (Tree Star Inc., Ashland OR) and Accuri cFlow plus software to obtain and confirm mean fluorescent intensity and proportion of positively expressed cells. Vehicle control was used to subtract for background noise and determine lipid uptake as a proportion of positive fluorescent signal and fluorescent signal intensity for all cells analyzed.

### 2.5 Inorganic nanopours vehicle synthesis

We prepared inorganic nanoporous vehicles according to the established methods [10, 11] using structure directing agents including the surfactant cetyltrimethylammonium bromide (CTAB) (1.17 g) dissolved in a solution containing water (180 mL) and ethylene glycol EG (30 mL) in an aqueous solution of ammonia (7.2 mL, 25 percent). TEOS (1.43 mL) and APS (0.264 mL) were rapidly added to the mixture after vigorous stirring for about 30 min at 323 K. In the solution, the final molar composition was 1 TEOS: 0.18 APS: 0.50 CTAB: 13.2 NH_3_: 84 EG: 1561.1H_2_O. The resulting mixture was stirred at 50 °C for another 2 h and then statically left for 20 h at the same temperature. We collected nanoporous vehicle samples with subsequent washing and redispersing steps by centrifugation at 14,000 rpm for 20 min. In a centrifuge tube, the synthesized nanoporous vehicles were dried at 60 °C. Then the nanoporous vehicle (1 g) and ammonium nitrate (NH_4_NO_3_) (0.3 g) were dissolved in ethanol (40 mL) and heated to extract the surfactant at 60 °C.

### 2.6 NOA synthesis

To functionalize the nanoporous vehicle samples, we prepared a mixture of APTES (40 μL) and nanoporous vehicle colloidal suspension (10 mg per 100 mL of ethanol) and refluxed it. After obtaining the aminated nanoporous vehicle samples, we integrated nanoporous vehicle with the optical nanoantenna structure names as nanoporous optical antenna (NOA). This was achieved by dropping nanoporous vehicle colloidal solution on a clean and hydrophilic SiO_2_ substrate, we immobilized nanoporous vehicles. After air drying, we deposited Au layer using the E-beam evaporation method (Angstrom Engineering Evovac Evaporator). During the Au deposition (deposition rate = 0.02 nm/s), we put the substrate above the Au sources with a certain tilt angle (∼60°) at a constant rotating speed (100 rpm). We isolated the Au coated nanoparticles from the SiO_2_ substrate by ultrasonic treatment for 2 h after completion of the deposition. The Au coated particles were then collected by centrifugation (∼10,000 rpm, 10 min) and suspended with ultrasonic treatment in DI water. The sample was then centrifuged and washed three to four times in water.

Adriamycin loading and release test: To prepare adriamycin-loaded NOA (A-NOA), we dispersed the prepared NOA in methanolic adriamycin solution (5 mg/mL). We stirred it overnight in the dark state to induce weak bonding between NOA and adriamycin. After removal the unloaded Adriamycin with centrifugation and washing steps, we dispersed A-NOA particles in a 1 mL buffer at different pH conditions from 7.4 to 6.5. The A-NOA particle solution was centrifuged at intervals of 1 h. The same volume of fresh buffers was added back to the residual mixture to wash the sample. By measuring the ultraviolet (UV) absorbance intensity of adriamycin in supernatant solution after centrifugation, we monitored drug release. The light-responsive properties were evaluated and the A-NOA was dispersed individually in buffer under light on (*λ* = 650 nm at ∼5 mW/cm^2^) and dark conditions. We monitored the release of light-dependent drugs by measuring the intensity of adriamycin after centrifugation in the supernatant solution (data not presented).

### 2.7 MTT assay

MCF-7 cells were seeded in a 96-well plate (0.32 cm^2^ growth area) at a density of 10^5^ cells per well and cultured to test the cytotoxicity of A-NOA and iNOVS. We then added them into the medium, respectively, for 72 h in 5% CO_2_ at 37 °C. At the end of the incubation, MTT solution (0.1 mg/mL) was added and incubated for another 4 h. The medium was then replaced with DMSO (50%) per well, and the absorbance was monitored using a microplate reader (Bio-TekELx800) at the wavelength of 595 nm. The cytotoxicity was expressed as the percentage of cell viability compared to untreated control cells. The optical density (OD) of the sample was measured at 570 nm with a microplate reader. The cytotoxicity (=(*Α* − *Β*)/*A* × 100, where *Α* is the absorbance of the cells incubated with the culture medium and *Β* is the absorbance of the cells incubated with the nanoparticles or the free drug).

### 2.8 Dark-field microscopy

For the intracellular adriamycin delivery, A-NOA particles (1 mg/1 mL) were internalized into MCF-7 cells which are placed in a petri dish. The cells are removed from the petri dish after 30 min, and are washed three times with 1X PBS. Afterward, the cell is mounted on a microscope slide and a PDMS micro chamber was placed on the microscope slide. The microscopy system consisted of an inverted microscope (Olympus IX73) equipped with a dark-field condenser (1.2–1.4 numerical aperture) and a white light source (Xenon Arc Lamp) to acquire images. Then, using a digital camera, the dark images of the treated cell and A-NOA were acquired (Q-color3, Olympus). A monochromator (Acton Research) with a cooled spectrograph coupled charge device (CCD) camera was used to gather scattering spectra from the samples at different locations (Roper Scientific). In front of the monochromator, we set up a 2 μm-wide aperture to hold only a single probe in the region of interest.

### 2.9 *In vitro* cell viability analyses

Cells were seeded in a 96-well plate at a density of 10^5^ cells per well and cultured in 5% CO_2_ at 37 °C for 48 or 72 h to test the cytotoxicity of SK101, SK-TS-101. Drugs were added into the medium for indicated amount of time. At the end of the incubation, 25 μL (MTS solution; Promega) was added and incubated for another 4 h. The medium was then replaced with 100 μL of dimethyl sulfoxide (DMSO) per well, and the absorbance was monitored using a microplate reader (Bio-TekELx800) at the wavelength of 595 nm. The cytotoxicity was expressed as the percentage of cell viability compared to untreated control cells. The optical density (OD) of the sample was measured at 570 nm with a microplate reader.

Calculation of drug sensitivity index for drug screening was achieved using MTS assay results as follows:

$$Drug\;sensitivity\;index\left(DSI\right)=\left[\frac{Average\;viability\;for\;drug\;‘X’\;in\;DTCCs\left(0.01,0.1,1.0,10\;\mu M\right)}{Average\;viability\;for\;drug\;‘X’\;in\;DNCCs\;\left(0.01,0.1,1.0,10\;\mu M\right)}\right]$$

Higher DSI=more effective kill in DTCCs

### 2.10 Synthesis of siRNA-plasmonic vehicles

Nanoparticles were prepared with similar protocol and concentrations as described previously [12]. Briefly, RNase-free gold nanorods (aspect ratio of 2.5 and absorbance of 1) were synthesized and prepared in 0.2 μm filtered DEPC-treated water. To remove excess CTAB, 500 μL gold nanorods were centrifuged and resuspended three times. To replace CTAB with cationic lipids, on the final centrifugation, a 10 μL pellet was resuspended in 50 μL of oligofectamine, briefly vortexed, and sonicated for 1 min. To conjugate NF-κΒ siRNA, 2 μL of 100 μM NF-κΒ siRNA was added to 500 μL of gold nanorods, vortexed and incubated for 30 min. To remove excess siRNA, gold nanorods washed by centrifugation, concentrated to a 10 μL pellet, and resuspended in 25 μL of oligofectamine.

### 2.11 Photoactivated release of siRNA from plasmonic vehicles

HeLa cells were cultured 20,000 cells/well in a 96-well plate for 24 h (0.32 cm^2^ growth area). HeLa cells were then washed once with Optimem media. A 0.5 μL concentrated pellet of siRNA-gold nanorods was added to 100 μL of Optimem media and added to the plated cells. After incubation for 4 h, the media was replaced with fresh supplemented DMEM culture media. The cells were then illuminated with 50 mW of a 660 nm CW diode laser, fluence = 0.64 W/cm^2^ (Newport Corp.) and incubated for 72 h. After 72 h, cells were harvested and fixed with cold 50% methanol for 3 min on ice followed by cold 100% methanol for 15 min on ice. Cells were immunostained with fluorescently labeled antibodies recognizing NF-κΒ and labeled with DAPI. Cells were imaged by fluorescence microscopy.

### 2.12 *In vivo* studies

Syngeneic mice model was generated using 4T1 breast cancer cells. Cells (1 × 10^6^) were implanted subcutaneously in the flanks of 5-week-old female BALB/c mice. Once the tumor size was 35 mm^3^, the mice were treated with vehicle or docetaxel (10 mg/kg) twice on alternate days. Further, depending on the treatment groups, the mice were treated everyday with vehicle, SK-101 (25 mg/kg) or SKTS-101 conjugate drug (25 mg/kg equivalent). The tumors were measured using a Vernier caliper, and tumor volume (*V*_t_) was calculated using the formula, *L* × W^2^/2, where *L* is the longest, and *W* is the shortest dimension. Tissues were harvested for further studies and the weight of the harvested tumors from each of the mice groups were also measured. All animal studies were performed under approved Institutional Use and Care of Animals Committee (IACUC) protocol at Harvard Medical School and Brigham and Women’s Hospital and handled in accordance with institutional guidelines.

### 2.13 Synthesis of SK-101 and SKTS-101

Please see Supplementary material for synthesis details.

## 3 Results

### 3.1 Case study 1: Lipid-targeted nanotherapeutics increase killing of drug-induced resistant cancer cells

#### 3.1.1 Screening lipid moieties that preferentially target drug tolerant cancer cells (DTCCs)

**Figure 1:**
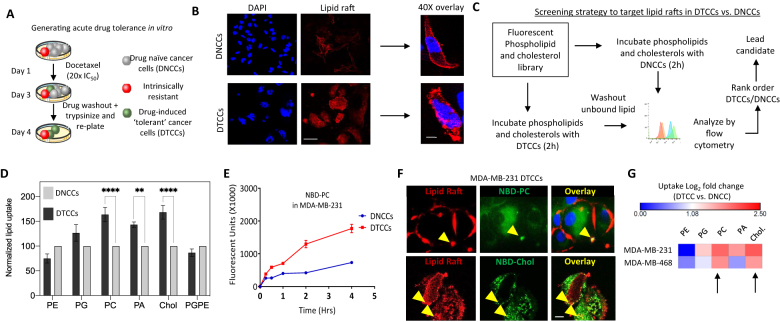
Lipid-based targeting of drug tolerant cancer cells (DTCCs). (A) Schematic representation of the experimental strategy for the generation of acute drug tolerant cancer cells (DTCCs) *in vitro*. (B) Representative florescent microscopy image of the lipid raft formation in drug naïve cancer cells (DNCCs) and DTCCs. Lipid raft labeling (red) and DAPI (blue). Scale bar = 10 μm right panel shows 20× image, scale bar = 3 μm. (C) The experimental workflow of the lipid screening strategy. (D) Histogram shows the normalized uptake of the lipid raft-targeting agents in DNCCs and in DTCCs. The fluorescent intensity obtained in each case has been recorded and normalized according to the DNCC fluorescence intensity. **p < 0.01 ****p < 0.0001 by *t*-test, *N* > 3 in biological replicate. (E) Trace shows uptake of NBD-PC [5 μM] into MDA-MB-231 DTCCs or DNCCs over time. Units are in arbitrary fluorescence as determined by flow cytometry, *N* = 3 in biological replicate. (F) Representative image showing the colocalization of lipid rafts and NBD-PC or NBD-cholesterol. DTCCs were incubated with NBD-PC/Cholesterol (green) and stained with lipid raft labeling agent red. These data demonstrates the internalization of the NBD-PC lipid across the lipid raft present on the cellular membrane performed in biological replicates. Scale bar = 10 μm. (G) Heatmap shows the change in fluorescence intensity of DTCCs relative to DNCCs from two TNBC cell lines (log_2_ fold change) as determined by flow cytometry. Arrows indicate lipids with increased uptake in DTCCs of both cell lines tested.

Cancer cells that have undergone acquired drug-induced resistance, or tolerance, can be collaterally sensitive to rationally-derived combination drug regimens [13, 14]. To optimize for a combination regimen in drug tolerant cancer cells, we deployed an *in vitro* model using the TNBC cell line, MDA-MB-231 [6]. Briefly, cells were exposed to a high dose of docetaxel; a taxane chemotherapy routinely used in first-line TNBC [15], and selected cells based on their capacity to readhere after acute population outgrowth. The persisting cells are referred to hereafter as drug tolerant cancer cells (DTCCs) (Figure 1A). We previously reported that DTCCs express a high concentration of plasma membrane lipid rafts compared to drug naïve cancer cells (DNCCs) [6]. Indeed, we confirmed this phenomenon using epifluorescent imaging of lipid rafts via detecting lipid raft bound cholera toxin (Figure 1B). Next, we developed a lipid-raft targeted screening protocol involving flow cytometry of fluorescently labeled lipids, which are characterized by different neutral or negative charges as well as unique log *P* values (Supplementary material Figure 1). Preferential binding and uptake into DTCCs was then evaluated (Figure 1C). Based on this screen, we determined that phosphatidylcholine (PC) and cholesterol resulted in significantly increased uptake into DTCCs vs. DNCCs and, to a lesser degree, phosphatidic acid (PA) at levels higher than the other lipids tested (Figure 1D). Indeed, we determined this effect was both dose and time dependent (Supplementary material Figure 2 and Figure 1E). We focused on PC and cholesterol and assessed binding onto lipid rafts of DTCCs using fluorescent staining and colocalization experiments (Figure 1F, yellow arrows). Finally, we determined that PC and cholesterol bound with the highest degree of specificity in multiple TNBC cell lines using epithelial-like MDA-MB-468 (Figure 1G, black arrows). Based on this information we concluded that either PC or cholesterol could function as a moiety to selectively target the induction of lipid rafts that develop on DTCCs.

#### 3.1.2 Identifying DTCC-sensitizing small molecule payloads

**Figure 2:**
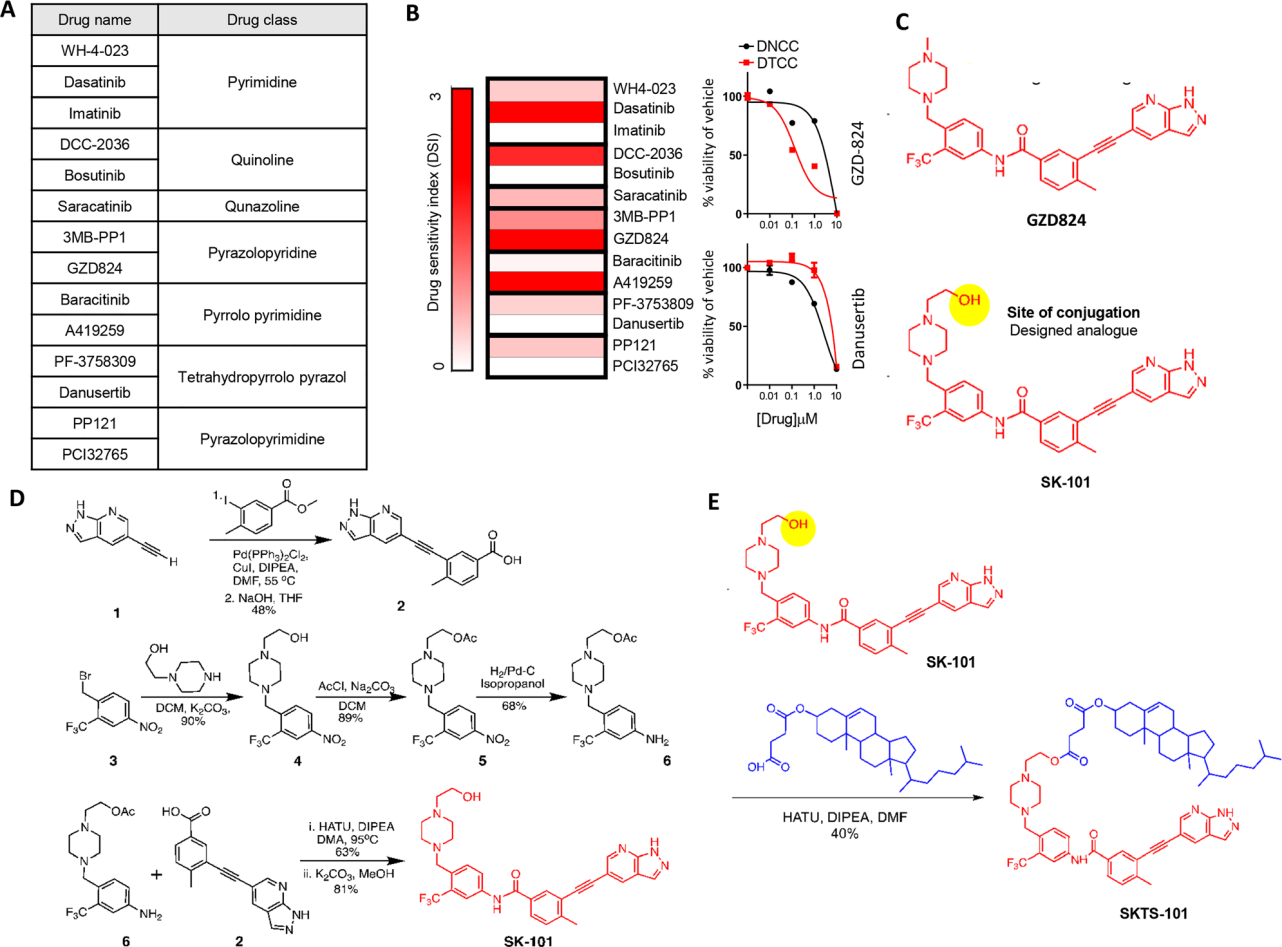
Selection of the drug candidate and introducing DTC targeting drug–lipid conjugate. (A) Collection of kinase inhibiting small molecules categorized into different drug classes depending on their chemical structure. (B) Heatmap representation of the drug sensitivity index (DSI) as determined in MDA-MB-231 cells. See Section 2 section for detailed calculation of DSI. Right panels show examples of high DSI (GZD824) and low DSI (Danusertib). (C) Structure of GZD824 and its modified analogue (SK-101). The modified analogue offers availability of site of conjugation where any targeting moiety can be attached highlighted in the bottom panel. (D) Synthetic scheme for SK-101. (E) Synthetic scheme of SKTS-101. Synthesis details in Supplementary material methods section.

Next, we wanted to engineer a therapeutic with a lipid-targeting moiety and a kinase-inhibiting payload to ‘home’ towards DTCCs and induce collateral sensitivity. Based on our previous evidence that SFK and HCK drive cell survival in DTCCs [6], we selected 14 drugs with published evidence for SFK or upstream kinase target affinities (Figure 2A). Next, we screened each drug based on a drug sensitivity index (DSI), which calculates a ratio of drug sensitivity in the DNCCs vs. DTCCs over a range of concentrations (see Section 2 for calculation); higher DSI indicates a greater selectively for DTCCs vs. DNCCs. Several molecules showed high selectivity for DTCCs including Dasatinib, DCC-2036, A419259 and GZD824 (Figure 2B and Supplementary material Figure 3). Indeed, while DCC-2036 and GZD824 were originally developed against BCR-Abl and variant mutations, they each display broad inhibition of SFK [16, 17] including putative Hck inhibition [18]. Notably, the pyrazolopyridine class (GZD824 and 3MB-PP1) was the only group of inhibitors to both show higher affinity for DTCCs vs. DNCCs with GZD824 displaying the highest level of specificity. Based on this information, we pursued GZD824 in subsequent experiments.

#### 3.1.3 Synthesizing a lipid-payload nanotherapeutic (SKTS-101)

To engineer the payload onto the lipid or cholesterol moiety, we first synthesized a novel molecule we termed SK-101, which has a similar structure to GZD824 but contains a terminal hydroxyl (–OH) group (see supplemental methods for chemical scheme). Based on published evidence, we determined that the hydroxyl group should not hinder the active site of SK-101 and thus offers an accessible chemical conjugation site to engineer a lipid-drug conjugate (Figure 2C). Following the detailed synthetic procedure (Figure 2D and Supplementary material) we engineered a conjugated product comprising the SK-101 payload onto the cholesterol moiety, which we refer to as, SKTS-101 (Figure 2E). This product was purified by column chromatography, characterized by ^1^H NMR, and used for further study (see Supplementary material).

#### 3.1.4 Testing *in vitro* and *in vivo* efficacy of SKTS-101

**Figure 3:**
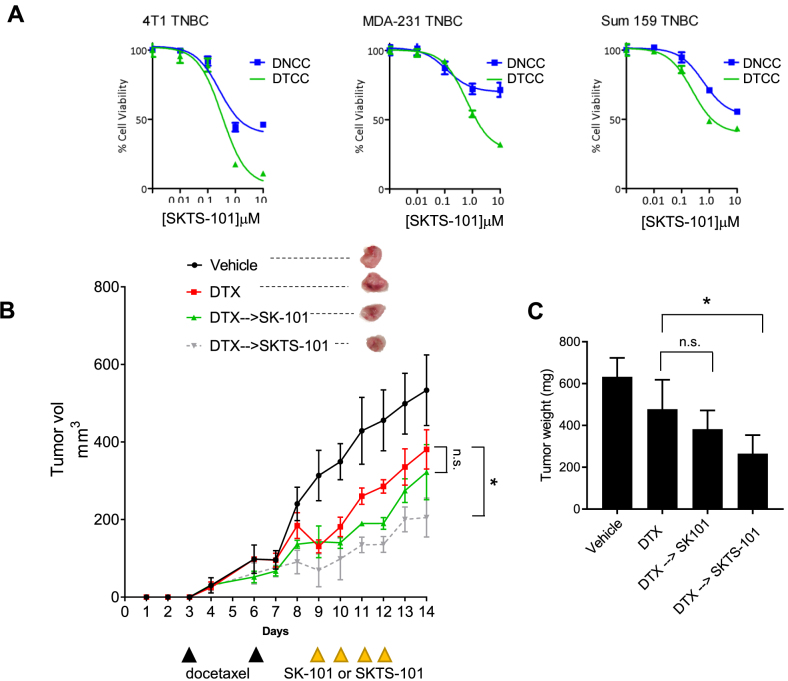
Validation of the efficacy of the novel lipid-drug conjugate *in vitro* and *in vivo*: (A) Cell viability analysis of SKTS-101 comparing efficacy in DTCCs and DNCCs from three different TNBC cell lines (4T1, MDA-MB-231 and SUM-159). (B) Tumor growth curve in 4T1 mammary carcinoma model with heterotopic implantation. The data are represented as mean ± SEM and statical analysis has been performed following unpaired t-test between the indicated groups on day 14. The upper inset shows representative images of the isolated tumors following sacrifice of the mice on day 14. *N* = 4 per group. (C) Bar plot showing the tumor mass comparing the tumor growth amongst the vehicle, DTX treated, SK-101 treated and the conjugated (SKTS-101) treated mice groups. The data represented as mean ± SEM and statical analysis has been performed following one-way ANOVA with a Dunnett’s multiple comparison test, where *p < 0.05.

First, we confirmed higher *in vitro* cytotoxicity of SKTS-101 in DTCCs vs. DNCCs confirmed by the separation in the kill curve using three TNBC cell lines: murine mammary 4T1, human MDA-MB-231 and SUM-159 (Figure 3A). Next, we established an *in vivo* 4T1 BALB/c syngeneic murine model to test the hypothesis that SKTS-101 will be more toxic in taxane-treated tumors due to the upregulation of lipid rafts and reliance on Hck [6]. Randomized cohorts were treated with a vehicle control, docetaxel (DTX), the free drug (SK-101), or conjugate drug (SKTS-101). We determined that SKTS-101 produced a statistically significantly slower tumor growth curve vs. DTX, while the free-drug, SK-101, did not (Figure 3B) (*p* < 0.05). These evidences were confirmed by analysis of tumor weight and visual inspection of tumor sizes after study termination (Figure 3C) (*p* < 0.05). Together, these findings support the design of rationally engineered nanotherapeutics that exploit cell surface properties of drug tolerant cancer cells to preferentially induce anticancer activity by signaling perturbations and collateral sensitivity.

### 3.2 Case study 2: Optically-responsive cancer nanoparticles for on-demand release of drug payloads that challenge resistance via endosomal recycling

#### 3.2.1 Proof of concept for on-demand optical release of payloads that circumvent endosomal recycling

**Figure 4:**
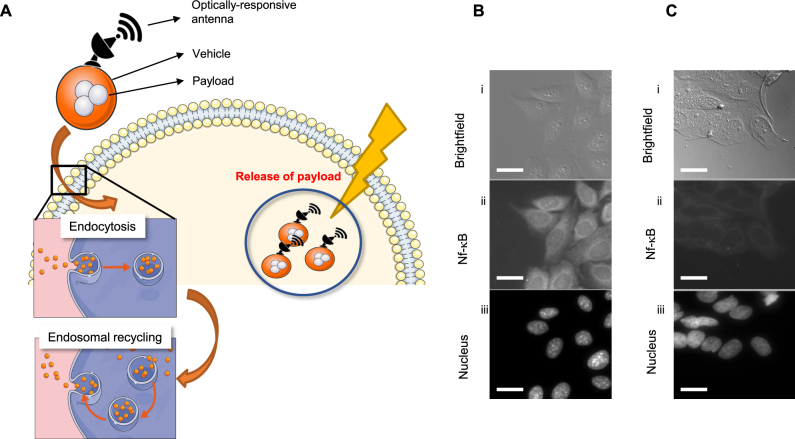
Photoactivated plasmonic nanoparticles as proof of concept on-demand release of payloads in tumor cells. (A) Schematic shows plasmonic nanoparticles as optical antennas and carriers of drug payloads that release under NIR. Inset shows hypothetical endosomal internalization of nanoparticles. (B and C) Representative images show NF-kB expression in HeLa cells treated with gold nanocarriers containing a control (scrambled) siRNA vector (b) or siRNA targeting the NF-kB gene (c) Panels show (i) DIC, (ii) anti NF-kB AF488 immunofluorescence, and (iii) DAPI. Scale bar = 10 μm.

Exocytosis of nanomedicines and endosomal recycling escape is a challenge-solution that is often considered during engineering of nanoparticle-based drugs [4]. This biological requires that nanoparticles retain properties to allow endosomal escape and release of therapeutic payloads within cells [8]. There are a number of ways this can be achieved with chemical engineering. One approach we hypothesize here is that plasmonics can be harnessed to preferentially engage nanoantenna using irradiation in the visible spectrum. Thus, circumventing resistance by exocytosis of nanocarriers, which we summarize schematically in (Figure 4A). We have previously developed on-demand optically addressable gold (Au) nanoantennas operating as optical receivers and biomolecular emitters of functionalized payloads [12]. In a first experiment, we deployed siRNA-Au nanoantennas as a proof of concept (PoC) to confirm plasmonic control and delivery of biomolecules. Using this approach as a positive control, we can show elimination of biological signals, on-demand. Gold nanorods of aspect ratios 2.5 and 4.0 were synthesized and coated by cationic lipids and loaded with siRNA that target the nuclear factor kappa B transcription factor (NF-kB). For the purposes of this PoC, we deployed an adenocarcinoma HeLa cell line and used a control experiment to show that unstimulated HeLa cells will produce high amounts of NF-kB, which represents ineffective disbursement of siRNA (Figure 4B). While nanoscale rods are highly endocytosed by cervical and adenocarcinoma cells such as HeLa cells, they also suffer from rapid clearance via exocytosis [19]. When nanorods were exposed to visible light (660 nm) stimulation, we confirmed the on-demand release of siRNA from the nanoroad, demonstrated by loss of fluorescent NF-kB antibody signal after treatment (Figure 4C). These data confirm that release of functionalized payloads can be controlled within tumor cells using plasmonics and potentially mitigating the exocytic function inherent in treatment of adenocarcinoma cells.

#### 3.2.2 Design of nanoporous optical antenna (NOA) with adriamycin

**Figure 5:**
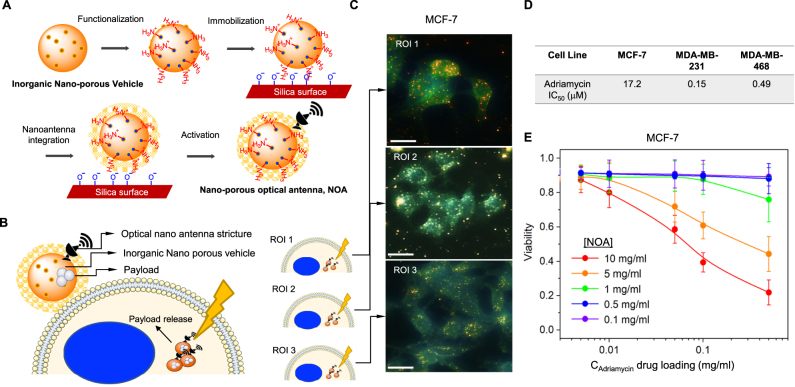
Photoactivated drug delivery from NOA. (A) Synthesis of optically responsive mesoporous silica nanoparticle by stepwise layer by layer coating method. (B) Schematic representation of the internalization of drug delivery by NOA in cancer cell. (C) Dark-field images of MCF7 cells with NOA. The internalized NOAs are shown as dots (scale bars = 20 μm). (D) Cell viability of MCF-7, MDA-MB-231 and MDA-MB-468 was assessed by MTT assay. IC_50_ was determined using a nonlinear curve fit analysis in GraphPad Prism. (E) Cell viability of MCF-7 cells after 24 h as a function of drug loading *C*_Adriamycin_ from 0.005 to 0.5 mg/mL at varied concentrations of NOA *C* = 0.1, 0.5, 1, 5, and 10 mg/mL.

Despite their ubiquitous and promising utility for cancer therapies, silica-containing nanoparticles represent nanomedicines that suffer from exocytic and endosomal recycling, which require the use of exocytosis-inhibiting or other agents [20]. Based on the PoC, above, we developed a plasmonic approach for inorganic nanoporous vehicles. We deployed a standard synthetic procedure that provides control of morphology, particle size, and uniformity [10]. CTAB was prepared in a solution containing water and EG followed by subsequent washing and redispersing steps by centrifugation. The synthesized inorganic nanoporous vehicles were dried, dissolved in ethanol and heated to extract the surfactant. These nanoporous optical antennas were then used to prepare nanoparticles capable of secondary functionalization via electron beam deposition for integration of optical nanoantenna structure, which we refer to hereafter as NOA (Figure 5A). Using adriamycin, an anticancer agent conventionally deployed for the treatment of luminal and triple-negative breast cancers for decades [21], we dispersed the aminated NOA with a methanolic adriamycin solution to induce weak bonding between NOA and adriamycin in the pore of the NOA. Notably, adriamycin is heavily associated with toxicity in breast cancer patients, which severely limits the use of these agents in the clinic despite putative benefits. We hypothesized that NOA could be therapeutically controlled through plasmonics to develop an on-demand and focal release of adriamycin, which should hypothetically mitigate exocytosis resistance mechanisms and therefore circumvent toxicity to normal systemic tissues and cells (Figure 5B).

#### 3.2.3 Photoactivated Adriamycin release and acute anticancer effects in MCF-7 breast cancer cells

Finally, we tested the anticancer efficacy of photoactivation in NOA. Darkfield images confirmed the internalization of drug-loaded nanoparticles into MCF-7 cells (Figure 5C). Initially, we wanted to test the relative resistance of cell lines against adriamycin. Using MCF-7, MDA-MB-231 and MDA-MB-468 TNBC cells, we evaluated cytotoxicity using cell viability analysis of a formazan dye conversion assay (MTT). We determined that MCF-7 are significantly more resistant to doxorubicin than either TNBC cell line as indicated by >5–10 fold shift in the IC_50_ (Figure 5D). Indeed, these data highlight the need to develop effective therapeutic tools to sensitize resistant cell lines. We loaded MCF-7 cells with increasing concentrations of NOA prior to the release of the drug by visible light illumination (*λ* = 650 nm). Using cell viability analysis via MTT, we confirmed dose-dependent induction of cell death could be controlled by the release of adriamycin from the NOA (Figure 5E). Taken together, these data confirm that functionalization of photoactivated payloads can be preferentially released to induce anticancer effects by NIR, thus circumventing physical barriers of resistance (i.e. endosomal recycling of nanoparticles).

## 4 Discussion

Bioengineering-based cancer therapies that can improve anticancer activity in tumors and preferentially target mechanisms of resistance is a final frontier in the quest for durable clinical responses. In this study, we leveraged discoveries in cancer biology and cancer drug resistance to facilitate the design of two distinct nanotechnology-based therapeutic tools. In the first example, we described how drug-induced resistance mechanisms can be exploited by engineered drug conjugates to deliver cell signaling disruptors that improve anticancer response. In the second example, we used nanophotonics to control the release of payloads, including cytotoxic drugs, in a manner that circumvents the physical barriers of exocytosis and endosomal recycling that plague bioengineered nanomedicines. Both examples highlight the need for innovation of translational tools that target resistance to increase efficacy of therapeutics for cancer.

In a previous report we described an induction and accumulation of lipid rafts in drug-induced resistant, or tolerant, cancer cells [6]. According to Lipinski rule the lipophilicity (log *P*) of a molecule controls the rate of penetration of a molecule through lipid bilayer [22]. Indeed, others have exploited this physical property for drug delivery [23]. Our approach was slightly different by optimizing lipophilicity for drug tolerant cells. An optimum lipophilicity, log *P* ∼ 5, helps the molecule to overcome the hydrophobic barrier of the phospholipid bilayer. As the DTCCs contain a higher degree of lipid rafts on the membrane, the lipophilic selection for the drugs is enhanced in case of membrane penetration. We described the degree of membrane penetration of PC, PA, and cholesterol is higher in case of DTCC than DNCC. Indeed, the log *P* values of PC, and cholesterol are close to 5 (6.15 and 7.25 respectively). Covalent conjugation of the PC lipid or cholesterol with the drug candidate may subsequently increase the penetration of the drug into DTCC cells because of favorable thermodynamic properties.

Nanophotonics for drug release and monitoring have been exploited [24]. However, several challenges associated with the previous approaches can be overcome by rationally designed therapeutics such as the NOA, which we describe here. For example, NOA enables better structural tunability, biocompatibility, and larger payload based on their highly ordered nanopore structure [25]. Through the integration of the optical nanoantenna structure we engineered here, our hybrid NOA enables a first-of-its-kind on-demand drug release feature that does not require internal enzymatic or pH-dependent gradients to dissolve the anticancer agent. Indeed, others have engineered thermo-responsive therapeutics [26], [27], [28], [29]. However, the tunable nature of NOA provides advantages for cancer drug delivery in focal areas of tumor growth under the skin that others have yet to be exploited.

Future design of nanophotonic-based anticancer drug designs that take advantage of discoveries in cancer and resistance biology are desperately needed. Indeed, our proof-of-concept study here opens the door to other inspired combinations of plasmonic based therapeutics, which exploit the cellular and molecular drivers of resistance. One example is the combination of both approaches we describe here to (1) engineer a chemotherapeutic regimen that focally releases anticancer cytotoxic drugs followed by (2) a second nanotherapeutic agent that preferentially eliminates the population of persisting drug-resistant cells. Such an approach could eliminate the pervasive occurrence of relapse that drives metastasis and mortality. More work is needed to bring these next generation approaches to fruition.

## Supplementary Material

Supplementary Material

Supplementary Material
