# Supplementary material for: Nanotherapeutic approaches to overcome distinct drug resistance barriers in models of breast cancer
Source: Nanophotonics. 2021 Jun 25;10(12):3063–73. doi: 10.1515/nanoph-2021-0142 (PMC8478290; doi:10.1515/nanoph-2021-0142)
Supplement: Supplementary file 1 — Supplementary Material [file j_nanoph-2021-0142_suppl_001.pdf]

Lipid raft-targeting agent	Structure	Characteristics (Type, Charge and logP)
Phosphatidylcholine (PC)	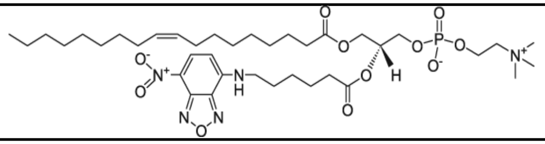	Phospholipid, Neutral, 6.15
Phosphatidylethanolamine(PE)	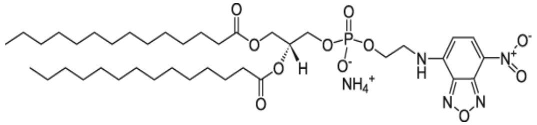	Phospholipid, Neutral, 8.67
Cholesterol	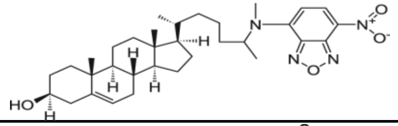	Triglycerides Lipid, Neutral, 7.25
Phosphatidic acid (PA)	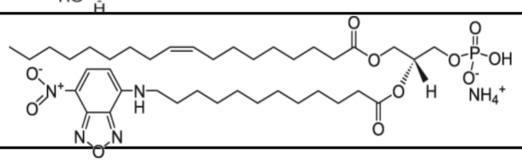	Phospholipid, Negative, 8.07
Phosphatidylserine (PS)	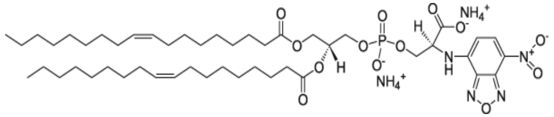	Phospholipids, Negative, 7.66
Phosphatidylglycerol (PG)	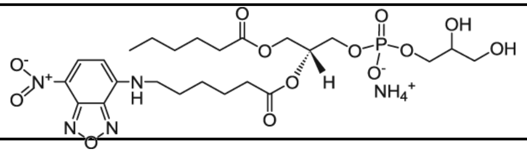	Glycerolipids, Negative, 1.86

Supplemental Figure 1: Structures of lipid used for the lipid screening assay

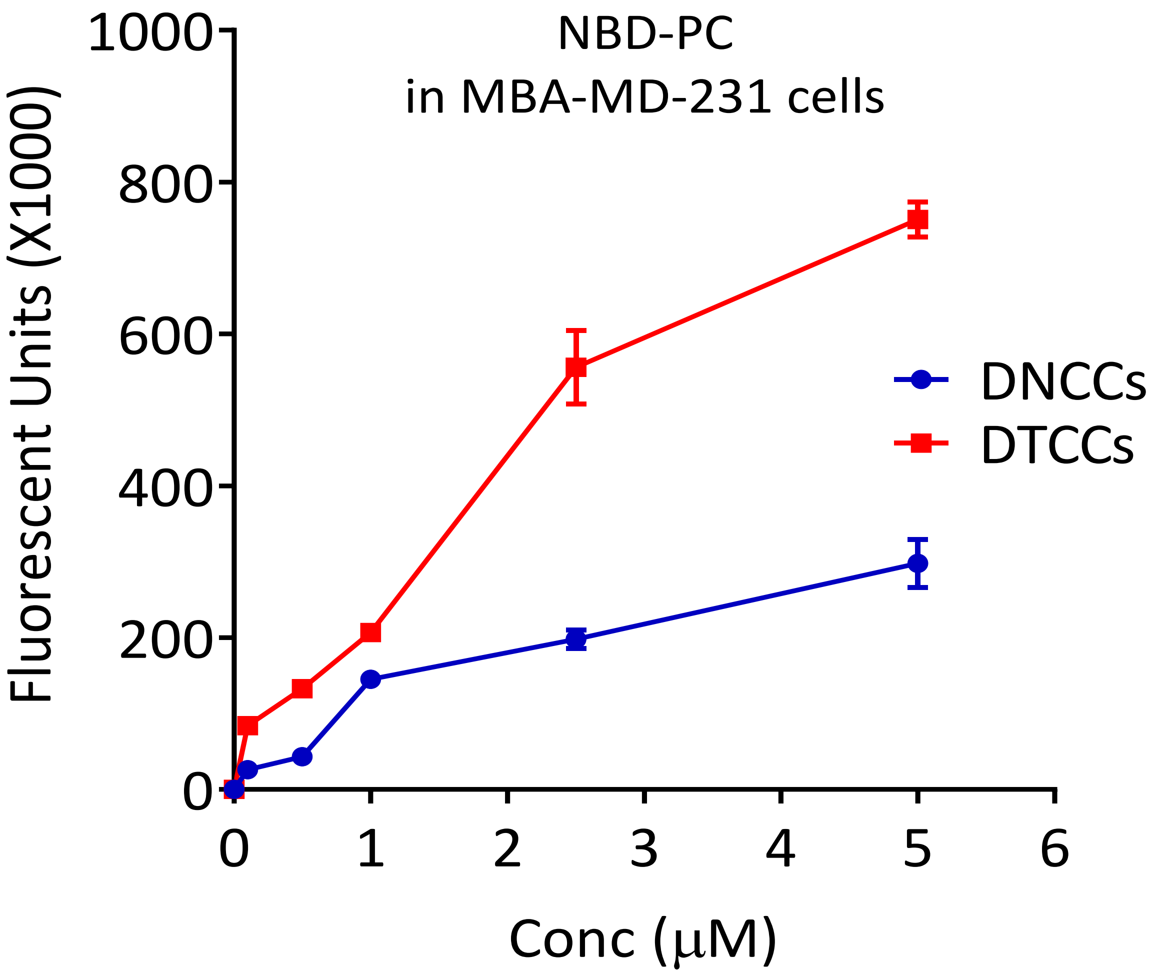

Supplemental Figure 2: Data represents kinetics of internalization of fluorescent lipid in DTCC and DNCC. NBD PC was added to the DNCC and DTCC and the amount of internalization has been checked at definite time interval. Data shows the higher rate of internalization of NBD-PC in case of DTCC.

Suppl. Fig 2 | Saha et al.

Drug	DNCCs [μM]	DTCCs [μM]
Dasatinib	8.6	0.11
A419259	7.1	0.83
Baracitinib	>1000	>1000
PF-3753809	4.0	2.8
3MB-PP1	>1000	16.0
GZD824	1.9	0.23
Imatinib	>1000	>1000
DCC-2036	24.9	0.519
PP121	2.7	.717
Bosutinib	3.4	40.6
Danusertib	1.9	4.2
Saracatinib	>1000	238.0
WH-4-023	29.0	19.1
PCI32765	>1000	>1000

Supplemental Figure 3: IC_{50} values calculated from cytotoxicity analysis of MDA-MB-231 cells.
