# Supplementary material for: Nanotherapeutic approaches to overcome distinct drug resistance barriers in models of breast cancer
Source: Nanophotonics. 2021 Jun 25;10(12):3063–73. doi: 10.1515/nanoph-2021-0142 (PMC8478290; doi:10.1515/nanoph-2021-0142)
Supplement: Supplementary file 2 — Supplementary Material [file j_nanoph-2021-0142_suppl_002.docx]

**Supplementary Information**

**Synthetic Procedure and Characterization Data for SKTS-101:**

**Synthesis of 3-[(1*H*-pyrazolo[3,4-b]pyridin-5-yl)ethynyl]-4-methylbenzoic acid (2):**

To a dry flask were added 5-ethynyl-1*H*-pyrazolo[3,4-b]pyridine (260 mg, 1.8 mmol), methyl 3-iodo-4-methylbenzoate (500 mg, 1.8 mmol), copper(I) iodide (35 mg, 0.18 mmol), and bis(triphenylphosphine)palladium(II) dichloride (63 mg, 0.09 mmol). The flask was evacuated and filled with nitrogen; and diisopropylamine (1 mL, 0.72 g, 7.2 mmol), and dry degassed DMF (10 mL) were added. Nitrogen was bubbled through the solution for 5 minutes. The reaction was then heated at 55°C for 8 h. The reaction was subsequently taken up in EtOAc (100 mL) and the solution was filtered through celite, and washed with sat. NH_4_Cl, sat. NaHCO_3_, and brine (100 mL each). The organic layer was dried over Na_2_SO_4_, and filtered. The volatiles were evaporated, and the residue was suspended in Et_2_O (50 mL) and stirred for 16 h. The suspension was filtered, and washed with additional Et_2_O. The solid was collected and dissolved in THF (50 mL) and 1 M NaOH (50 mL), and stirred for 8 h. The solution was then cooled in an ice-bath and acidified by addition of 1 N HCl. The precipitate was filtered, collected, and washed with cold EtOAc, and Et_2_O, and dried under vacuum. The product, 3-[(1*H*-pyrazolo[3,4-b]pyridin-5-yl)ethynyl]-4-methylbenzoic acid (**2**), was obtained without the need for further purification in 48% yield (240 mg, 0.86 mmol), over 2 steps.

**^1^H NMR** (400 MHz, DMSO-d6): δ = 2.55 (s, 3 H), 7.46 (d, *J* = 8.0 Hz, 1 H), 7.84 (d, *J* = 8.0 Hz, 1 H), 8.05 (s, 1 H), 8.20 (s, 1 H), 8.50 (s, 1 H), 8.71 (s, 1 H).

**Synthesis of 2-{4-[4-nitro-2-(trifluoromethyl)benzyl]piperazin-1-yl}ethan-1-ol (3):**

To a dry flask were added 1-(bromomethyl)-4-nitro-2-(trifluoromethyl)benzene (500 mg, 1.8 mmol), K_2_CO_3_ (243 mg, 1.8 mmol), and dry DCM (10 mL). The solution was cooled in an ice-bath, and 2-(piperazin-1-yl)ethan-1-ol was added dropwise. (260 mg, 0.26 mL, 2.0 mmol). The reaction was allowed to stir for 8 h under nitrogen. The reaction was again cooled in ice, filtered, and volatiles were evaporated. The residue was purified by silica gel chromatography, using a gradient of DCM:MeOH:TEA (99:0:1 to 90:9:1). The product **3** was obtained in 90% yield (540 mg, 1.62 mmol).

**Synthesis of 2-{4-[4-nitro-2-(trifluoromethyl)benzyl]piperazin-1-yl}ethyl acetate (4):**

To a dry flask were added 2-{4-[4-nitro-2-(trifluoromethyl)benzyl]piperazin-1-yl}ethan-1-ol (**2**) (540 mg, 1.62 mmol), and K_2_CO_3_ (243 mg, 1.8 mmol), and dry DCM (10 mL). The solution was cooled in an ice-bath, and acetyl chloride (127 mg, 0.11 mL, 1.62 mmol) was added dropwise to the solution. The reaction was allowed to stir for 8 h under nitrogen. The reaction was again cooled in ice, filtered, and washed with sat. NaHCO_3_ (10 mL), and brine (10 mL). The organic layer was dried over Na_2_SO_4_, and filtered. The volatiles were evaporated. The residue was purified by silica gel chromatography, using a gradient of DCM:MeOH:TEA (99:0:1 to 90:9:1). The product **3** was obtained in 89% yield (525 mg, 1.4 mmol).

**^1^H NMR** (400 MHz, CDCl_3_): δ = 2.04 (s, 3 H), 2.51-2.55 (m, 8 H), 2.63 (t, *J* = 6.0 Hz, 2 H), 3.72 (s, 2 H), 4.18 (t, *J* = 6.0 Hz, 2 H), 8.08 (d, *J* = 8.8 Hz, 1 H), 8.35 (d, *J* = 8.4 Hz, 1 H), 8.47 (s, 1 H).

**Synthesis of 2-{4-[4-amino-2-(trifluoromethyl)benzyl]piperazin-1-yl}ethyl acetate (4):**

To a flask containing 2-{4-[4-nitro-2-(trifluoromethyl)benzyl]piperazin-1-yl}ethyl acetate (**3**) (525 mg, 1.4 mmol) were added degassed EtOAc (5 mL), degassed isopropanol (5 mL), and Pd/C (10% wt.) (250 mg). The flask was cooled, evacuated and the atmosphere replaced with H_2_. The reaction was stirred at rt, and monitored for completion by LC/MS (about 6 h). The reaction was filtered through celite, and the volatiles were evaporated. The residue was further purified by silica gel chromatography , using a gradient of DCM:MeOH:TEA (99:0:1 to 90:9:1). The product **4** was obtained in 68% yield (330 mg, 0.95 mmol).

**^1^H NMR** (400 MHz, CDCl_3_): δ = 2.04 (s, 3 H), 2.44-2.53 (m, 8 H), 2.62 (t, *J* = 6.0 Hz, 2 H), 3.51 (s, 2 H), 3.76 (br s, 2 H), 4.18 (t, *J* = 6.0 Hz, 2 H), 6.77 (d, *J* = 8.4 Hz, 1 H), 6.90 (s, 1 H), 7.44 (d, *J* = 8.4 Hz, 1 H). LRMS: *m/z* calcd for C_16_H_22_F_3_N_3_O_2_ [M+H]^+^: 346.2; found: 346.2.

**Synthesis of 2-[4-(4-{3-[(1*H*-pyrazolo[3,4-b]pyridin-5-yl)ethynyl]-4-methylbenzamido}-2-(trifluoromethyl)benzyl)piperazin-1-yl]ethyl acetate (5):**

To a dry flask were added carboxylic acid **2** (120 mg, 0.43 mmol), aniline **4** (150 mg, 0.43 mmol), and HATU (181 mg, 0.47 mmol). The flask was put under N_2_ and dry DMA (3 mL) and dry DIPEA (122 mg, 0.17 mL, 0.95 mmol) were added. The reaction was heated at 95°C for 8 h. The reaction was then taken up in EtOAc (50 mL) and washed with sat. NaHCO_3_ (50 mL), and brine (2 × 50 mL). The organic layer was dried over Na_2_SO_4_, and filtered. The volatiles were evaporated. The residue was purified by silica gel chromatography, using a gradient of DCM:MeOH:TEA (99:0:1 to 90:9:1). The product **5** was obtained in 63% yield (165 mg, 0.27 mmol).LRMS: *m/z* calcd for C_32_H_31_F_3_N_6_O_3_ [M+H]^+^: 605.2; found: 605.2.

**Synthesis of 3-[(1*H*-pyrazolo[3,4-b]pyridin-5-yl)ethynyl]-*N*-(4-{[4-(2-hydroxyethyl)piperazin-1-yl]methyl}-3-(trifluoromethyl)phenyl)-4-methylbenzamide (6):**

To a dry flask were added intermediate **5** (165 mg, 0.27 mmol), K_2_CO_3_ (15 mg, 0.1 mmol), and dry MeOH (3 mL). The reaction was stirred for 8 h at rt. The reaction was filtered, and the volatiles evaporated. The residue was purified by silica gel chromatography, using a gradient of DCM:MeOH:TEA (99:0:1 to 85:14:1). The product **SK-101** was obtained in 81% yield (122 mg, 0.22 mmol).

LRMS: *m/z* calcd for C_30_H_29_F_3_N_6_O_2_ [M+H]^+^: 563.2; found: 563.2.

**1H NMR:** (400 MHz, DMSO-d6): δ 10.55 (s, 1H), 8.74 (d, J = 2.0 Hz, 1H), 8.53 (d, J = 2.0 Hz, 1H), 8.26 ñ 8.18 (m, 3H), 8.07 (dd, J = 8.5, 2.2 Hz, 1H), 7.93 (dd, J = 8.0, 2.0 Hz, 1H), 7.71 (d, J = 8.5 Hz, 1H), 7.53 (d, J = 8.1 Hz, 1H), 4.35 (s, 1H), 3.56 (s, 2H), 3.48 (d, J = 4.2 Hz, 2H), 2.59 (s, 3H), 2.38 (t, J = 6.4 Hz, 9H).

**13C NMR** (100 MHz, DMSO-d6): δ 164.70, 151.00, 132.98, 132.11, 131.26, 130.57, 129.92, 123.52, 122.16, 113.99, 111.76, 91.90, 88.27, 79.19, 20.43.

**Synthesis of** **2-(4-(4-(3-((1*H*-pyrazolo[3,4-*b*]pyridin-5-yl)ethynyl)-4-methylbenzamido)-2-(trifluoromethyl)benzyl)piperazin-1-yl)ethyl (10,13-dimethyl-17-(6-methylheptan-2-yl)-2,3,4,7,8,9,10,11,12,13,14,15,16,17-tetradecahydro-1*H*-cyclopenta[*a*]phenanthren-3-yl) succinate:**

To a clean and dry flask were added compound **6** (30 mg, 0.053 mmol), DIPEA (0.212 mmol), HATU ( 0.1 mmol) and DMF. The reaction mix was stirred for 10 min at room temp under inert atmosphere. Cholesteryl hemisuccinate (0.08 mmol) and the mixture were stirred for 24h. The reaction was monitored by TLC and the expected compound was purified by silica gel chromatography, using a gradient of DCM:MeOH: (100:0 to 90:10). The final product **SKTS-101** was obtained in 40% yield (21.8 mg, 0.02 mmol).

**^1^H NMR** (400 MHz, Chloroform-*d*): δ 8.74 (d, *J* = 1.9 Hz, 1H), 8.29 (d, *J* = 1.9 Hz, 1H), 8.06 – 7.99 (m, 1H), 7.97 (s, 1H), 7.89 (d, *J* = 9.5 Hz, 1H), 7.83 – 7.73 (m, 2H), 7.70 (dd, *J* = 5.7, 3.3 Hz, 1H), 7.53 (dd, *J* = 5.7, 3.3 Hz, 1H), 7.40 (d, *J* = 8.1 Hz, 1H), 5.35 (s, 1H), 4.59 (s, 1H), 4.23 (d, *J* = 28.6 Hz, 3H), 3.66 (s, 2H), 3.15 (s, 1H), 2.75 (s, 2H), 2.70 – 2.53 (m, 8H), 2.31 (d, *J* = 7.3 Hz, 2H), 1.85 (d, *J* = 12.1 Hz, 4H), 1.64 – 1.17 (m, 26H), 1.01 (s, 3H), 0.88 (m, 14H). **^13^C NMR** (100 MHz, DMSO-D6): δ 209.53, 203.20, 196.33, 165.19, 155.83, 151.48, 145.32, 144.18, 142.94, 138.85, 133.46, 132.59, 131.74, 131.05, 130.40, 128.64, 128.64, 127.74, 124.00, 123.45, 122.64, 114.47, 112.23, 92.37, 88.74, 79.66, 57.45, 52.70, 49.03, 44.56, 37.12, 20.90.
